# Associations of Bioavailable Serum Testosterone With Cognitive Function in Older Men: Results From the National Health and Nutrition Examination Survey

**DOI:** 10.1093/gerona/glac162

**Published:** 2022-08-05

**Authors:** Panagiotis Giannos, Konstantinos Prokopidis, David D Church, Ben Kirk, Paul T Morgan, Mary Ni Lochlainn, Helen Macpherson, David R Woods, Theocharis Ispoglou

**Affiliations:** Department of Life Sciences, Faculty of Natural Sciences, Imperial College London, London, UK; Society of Meta-research and Biomedical Innovation, London, UK; Society of Meta-research and Biomedical Innovation, London, UK; Department of Musculoskeletal Biology, Institute of Life Course and Medical Sciences, University of Liverpool, Liverpool, UK; Department of Geriatrics, Donald W. Reynolds Institute on Aging, Center for Translational Research in Aging and Longevity, University of Arkansas for Medical Sciences, Little Rock, Arkansas, USA; Department of Medicine-Western Health, Melbourne Medical School, University of Melbourne, Melbourne, Victoria, Australia; Australian Institute for Musculoskeletal Science (AIMSS), University of Melbourne and Western Health, St. Albans, Melbourne, Victoria, Australia; School of Sport, Exercise and Rehabilitation Sciences, University of Birmingham, Birmingham, UK; Department of Sport and Exercise Sciences, Manchester Metropolitan University, Manchester, UK; Department of Twin Research and Genetic Epidemiology, King’s College London, London, UK; Deakin University, Geelong, Victoria, Australia; Institute for Physical Activity and Nutrition (IPAN), School of Exercise and Nutrition Sciences, Burwood, Victoria, Australia; Defence Medical Services, Lichfield, UK; Carnegie School of Sport, Leeds Beckett University, Leeds, UK; Carnegie School of Sport, Leeds Beckett University, Leeds, UK

**Keywords:** Aging, Bioavailable testosterone, Cognition, Dementia, Older men

## Abstract

**Background:**

Age-associated cognitive decline may be influenced by testosterone status. However, studies evaluating the impact of bioavailable testosterone, the active, free testosterone, on cognitive function are scarce. Our study determined the relationship between calculated bioavailable testosterone and cognitive performance in older men.

**Methods:**

We used data from the U.S. National Health and Nutrition Examination Survey (NHANES) between 2013 and 2014. This study consisted of 208 men aged ≥60 years. Bioavailable serum testosterone was calculated based on the total serum testosterone, sex hormone–binding globulin, and albumin levels, whereas cognitive performance was assessed through the Consortium to Establish a Registry for Alzheimer’s Disease (CERAD) Word List Learning Test (WLLT), Word List Recall Test (WLRT), and Intrusion Word Count Test (WLLT-IC and WLRT-IC), the Animal Fluency Test (AFT), and the Digit Symbol Substitution Test (DSST). Multiple linear regression analyses were performed upon adjustment for age, ethnicity, socioeconomic status, education level, medical history, body mass index, energy, alcohol intake, physical activity levels, and sleep duration.

**Results:**

A significant positive association between bioavailable testosterone and DSST (β: 0.049, *p* = .002) score was detected, with no signs of a plateau effect. No significant associations with CERAD WLLT (*p* = .132), WLRT (*p* = .643), WLLT-IC (*p* = .979), and WLRT-IC (*p* = .387), and AFT (*p* = .057) were observed.

**Conclusion:**

Calculated bioavailable testosterone presented a significant positive association with processing speed, sustained attention, and working memory in older men above 60 years of age. Further research is warranted to elucidate the impact of the inevitable age-related decline in testosterone on cognitive function in older men.

Age-related cognitive decline can be a precursor of dementia, which currently remains a major public health challenge (1). An aging brain is characterized by a reduction in gray and white matter volume and is associated with decreased processing, attention, memory, and executive function (2). In the United States, there are ~5.1 million people living with dementia (3), with an estimated financial impact projected at >$9 trillion worldwide by 2050 (4). Currently, there are multiple risk factors associated with cognitive decline throughout the lifespan, including genetic predisposition, malnutrition, physical inactivity, and androgen deficiency (5,6), factors that represent an important focus for future research in an attempt to treat and/or manage a cognitive decline in older age.

Evidence regarding the influence of the primarily male hormone testosterone on cognitive function is conflicting (7–13), while both total and bioavailable levels of testosterone consistently decline with aging (14). Specifically, previous studies have observed associations between low serum testosterone levels and decreased cognitive performance (7,8) or dementia severity (9), whereas other studies have neither observed associations (10,13) nor negative correlations between these variables (11,12). These findings have led to uncertainty as to whether serum testosterone is linked with risk of cognitive decline in men during aging.

One of the challenges in interpreting published literature is that studies variably report total testosterone or bioavailable testosterone. The majority of testosterone within the human body is tightly bound (~60% of total testosterone) to sex hormone–binding globulin (SHBG) and to a lesser extent (~38%) bound to albumin. Only a small fraction (~2%) of total testosterone is unbound or “free” and thus “biologically active” and available at the tissue level (15). SHBG may vary according to a wide variety of factors including nutritional state, weight, androgen levels, intercurrent illness, and age (16,17). The subsequent influence this has on testosterone binding and thus activity makes bioavailable testosterone a more preferable parameter than total testosterone in isolation. Indeed, as total testosterone typically reduces with age, while SHBG typically increases (18), the overall effect of aging will have a greater effect on bioavailable, active testosterone. Therefore, the aim of the current cross-sectional study was to examine the association of bioavailable testosterone with cognitive function in older men.

## Method

### Study Design and Participants

We retrieved publicly available data from participants aged ≥60 years from one survey cycle in the National Health and Nutrition Examination Survey (NHANES): 2013–2014. NHANES is conducted by the Centers for Disease Control and Prevention (CDC) and the National Center for Health Statistics (NCHS) to monitor health in the U.S. population. A cutoff age of ≥60 years old was used based on data availability for total serum testosterone, SHBG, and albumin concentrations. Exclusion criteria included no recorded data for total serum testosterone, SHBG, and albumin concentrations, and incomplete data for the cognitive assessments or missing demographic data. The NHANES protocol was approved by the NCHS Research Ethics Review Board, while all participants provided written informed consent.

### Bioavailable Testosterone Assessment

Bioavailable serum testosterone was computed according to the Vermeulen methodology (19) using measured concentrations of total serum testosterone, SHBG, and albumin. Total serum testosterone from overnight fasted samples was estimated through isotope dilution–liquid chromatography–tandem mass spectrometry (ID–LC–MS/MS) method. SHBG was reacted with immune antibodies and chemoluminescence measurements of microparticles and measured by a photomultiplier tube. Albumin concentration was assessed using the DcX800 method by means of a biochromatic digital endpoint methodology with Bromcresol Purple.

### Cognitive Assessment

Cognitive function was evaluated using a variety of tests including the Consortium to Establish a Registry for Alzheimer’s Disease (CERAD) Word List Learning Test (WLLT), Word List Recall Test (WLRT), and Intrusion Word Count Test (WLLT-IC and WLRT-IC), the Animal Fluency Test (AFT), and the Digit Symbol Substitution Test (DSST). The assessments were administered by trained, qualified personnel at the end of the in-person private interview at the mobile examination centers. The full details of the cognitive function interviews have been presented elsewhere (https://www.cdc.gov/nchs/nhanes/index.htm).

The CERAD WLLT, WLLT-IC, WLRT, and WLRT-IC examine the immediate and delayed learning ability for novel verbal information and consist of 3 progressive learning trials followed by a delayed recall challenge with a range of scores between 0 and 10. The AFT assesses executive function by evaluating categorical verbal fluency with scores ranging from 3 to 39. The DSST comprises a performance challenge from the Wechsler Adult Intelligence Scale-III, which assays processing speed, sustained attention, and working memory, and is scored between 0 and 105. Higher test scores depict better cognitive performance. Participants without a response for any of the tests were excluded.

### Covariates

Age (years), ethnicity (race), socioeconomic status (family income to poverty ratio [FIPR]), education level (school qualification), medical history (memory–cognitive function loss and stroke), body mass index (BMI; kg/m^2^), daily sleep duration (hours of sleep spent at night on weekdays or workdays) and physical activity (minutes spent doing moderate-intensity sports, fitness, or recreational activities), daily energy intake (kcal), and alcohol intake (g) were considered as covariates. All covariates were identified as potential confounders in the relationship between bioavailable testosterone and cognitive performance. Participants with a current medical prescription of aromatase inhibitors and glucocorticoids were excluded, considering that they may alter endogenous testosterone levels (20).

Age groups consisted of participants with ≥60 years of age and classified into 60–69, 70–79, and ≥80 years of age. Ethnic groups comprised of Mexican American, other Hispanic, non-Hispanic White, non-Hispanic Black, non-Hispanic Asian, and other (multi) race. Social economic status was categorized as low–middle (FIPR < 1) and middle–high (FIPR ≥ 1). Education level was defined as no high school degree, at most a high school degree or a college degree at minimum. Medical history based on loss of cognitive-memory function or stroke was categorized as Yes/No responses in terms of past incidence reported by a doctor or other health professional. BMI was defined as a participant’s weight in kilograms divided by the square of height in meters. A physical activity of <150 min/wk was considered low-moderate and ≥150 min/wk was considered moderate-high. Energy and alcohol intake were calculated as averages of the 24-hour dietary recall and categorized into low, moderate, and high. A BMI of <18 kg/m^2^ was considered low, 18–24.9 kg/m^2^ moderate and ≥25 kg/m^2^ high. An energy intake of <2 000 kcal was considered as low, 2 000–3 000 kcal moderate, and >3 000 kcal high. Alcohol intake < 15 g was considered low, 15–30 g moderate, and >30 g high. Sleep duration of ≤6 hours was classified as low, 7–9 hours as moderate, and >9 hours as high.

### Statistical Analysis

Multiple linear regression analyses were performed to examine the association between bioavailable and total testosterone and cognitive function (overall and test-specific cognitive performance) with the adjustment of all covariates. A restricted cubic spline was employed to model the nonlinear and dose–response relationship between calculated bioavailable testosterone and cognitive function using 3 knots after covariate adjustments. Statistical significance was established as *p* < .05. Statistical analysis was performed using IBM SPSS statistics software (Version 28.0, IBM Corp., Armonk, NY).

## Results

### Characteristics of Study Participants

**Figure 1.**
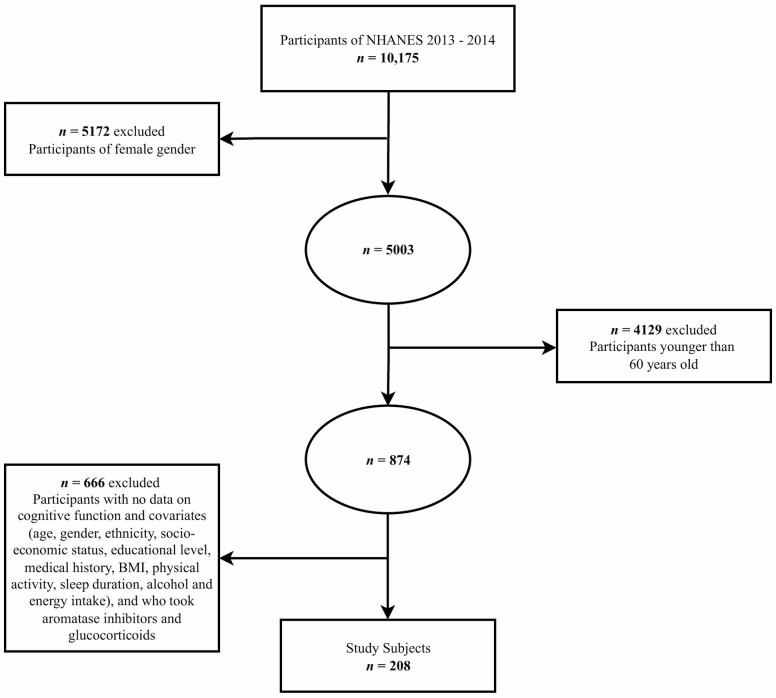
Flow chart of the screening process for the selection of eligible participants in the National Health and Nutrition Examination Survey (NHANES).

Data for cognitive function and calculated bioavailable testosterone were available for a total of 208 participants (Figure 1). Background information (ie, sociodemographic status, anthropometrics, and nutritional characteristics) of all participants are reported in Table 1. The study population had a mean age of 69.4 (± 0.5) years and consisted of mostly non-Hispanic White (53%) of high socioeconomic status (69%) with a college degree at minimum (67%). Sleep duration was moderate (63%) among participants and physical activity was high (55.3%). Energy intake was below the typical recommended consumption (51%) or within recommendations (42%). Alcohol intake was low (78%), and BMI was high (71%). The mean calculated bioavailable testosterone was 185.7 (± 4.0) ng/dL. The average score for CERAD WLLT was 19.3 (± 0.3) of 30, 6.1 (± 0.2) of 10 for CERAD WLRT, 0.6 (± 0.1) of 12 for CERAD WLLT-IC, 0.4 (± 0.1) of 10 for CERAD WLRT-IC, 18.3 (0.4) of 40 for the AFT, and 49.0 (± 1.0) of 100 for the DSST.

**Table 1.** Sociodemographic, Behavioral, and Nutritional Characteristics of Included Participants (*n* = 208)

Age	
60–69	109 (52)
70–79	66 (32)
≥80	33 (17)
Ethnicity	
Mexican American	22 (11)
Other Hispanic	15 (7)
Non-Hispanic White	111 (53)
Non-Hispanic Black	38 (18)
Non-Hispanic Asian	19 (9)
Other Race—including multiracial	3 (1)
Socioeconomic status	
Low	17 (8)
Middle	47 (23)
High	144 (69)
Educational level	
No high school degree	31 (15)
High school degree	38 (18)
College degree	139 (67)
Energy intake	
Low	105 (51)
Moderate	88 (42)
High	15 (7)
Body mass index	
Low	2 (1)
Normal	59 (28)
High	147 (71)
Alcohol intake	
Low	163 (78)
Moderate	20 (10)
High	25 (12)
Physical activity	
Low	30 (14)
Moderate	63 (30)
High	115 (55)
Sleep duration	
Low	56 (27)
Moderate	130 (63)
High	22 (11)
Bioavailable serum testosterone (ng/dL)	
Minimum	22.9
Average	185.7 (12.0)
Maximum	543.4
Total serum testosterone (ng/dL)	
Minimum	87.8
Average	415.0 (12.5)
Maximum	1 260.0
CERAD WLLT (score)	
Minimum	0.0
Average	19.3 (0.3)
Maximum	30.0
CERAD WLRT (score)	
Minimum	0.0
Average	6.1 (0.2)
Maximum	10.0
CERAD WLLT-IC (score)	
Minimum	0.0
Average	0.6 (0.1)
Maximum	6.0
CERAD WLRT-IC (score)	
Minimum	0.0
Average	0.4 (0.1)
Maximum	4.0
AFT (score)	
Minimum	7.0
Average	18.3 (0.4)
Maximum	36.0
DSST (score)	
Minimum	11.0
Average	49.0 (1.0)
Maximum	93.0

*Notes:* AFT = Animal Fluency Test; CERAD = Consortium to Establish a Registry for Alzheimer’s Disease; DSST = Digit Symbol Substitution Test; WLLT = Word List Learning Test; WLRT = Word List Recall Test. WLLT-IC = Word List Learning Test—Intrusion Word Count; WLRT-IC = Word List Recall Test—Intrusion Word Count. Values are expressed as count (percentage) unless otherwise specified.

### Calculated Bioavailable Testosterone and Cognitive Function

**Figure 2.**
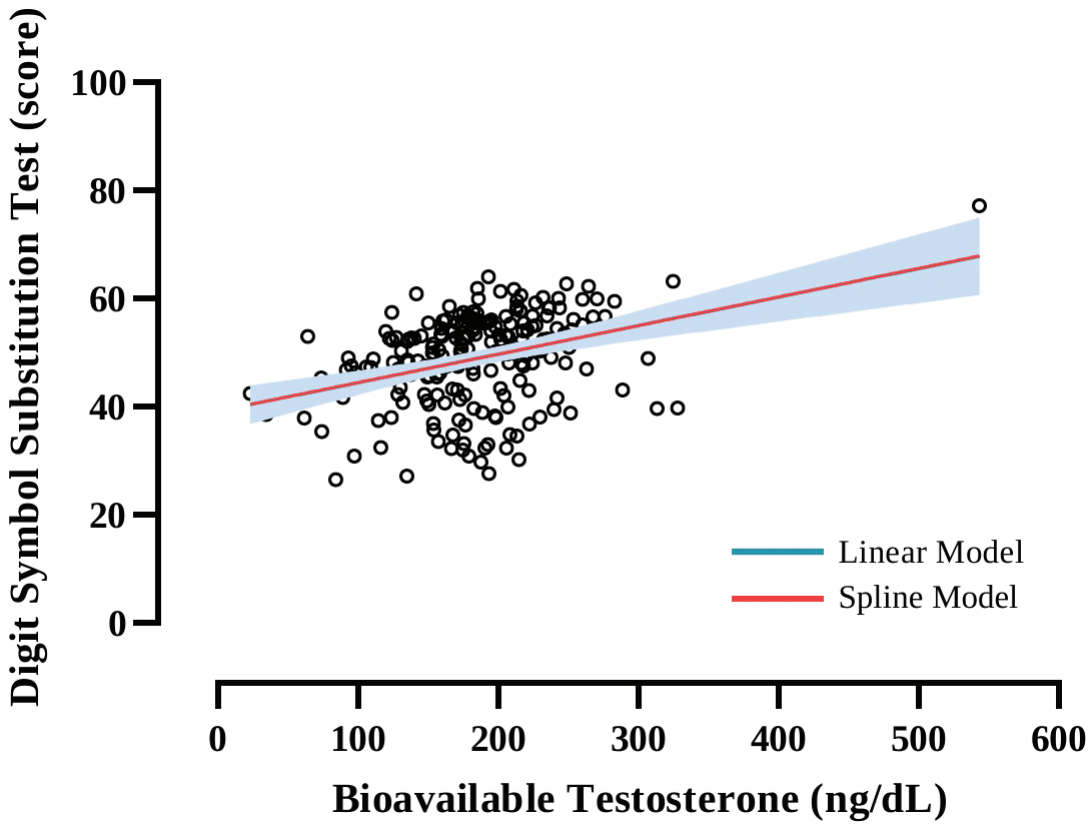
Dose–response relationship between bioavailable serum testosterone and cognitive function in older male adults. Significant association with Digit Symbol Substitution Test score was observed. Multiple linear and cubic-spline models were adjusted for age, gender, ethnicity, socioeconomic status, educational level, medical history, body mass index, physical activity, sleep duration, alcohol, and energy intake.

Calculated bioavailable testosterone was significantly positively associated with the DSST (β: 0.049, *p* = .002) score (Table 2). No significant associations were found with CERAD WLLT (*p* = .132), WLRT (*p* = .643), WLLT-IC (*p* = .979), and WLRT-IC (*p* = .387), and AFT (*p* = .057). Cubic spline modeling showed no signs of a plateau effect across the distribution of calculated bioavailable testosterone levels explored on the significant associations observed (Figure 2). According to our multiple linear regression model, a change in bioavailable testosterone of 100 ng/dL corresponded to a 5% improvement in DSST performance. Finally, similar analysis of the same cohort based on total testosterone concentrations showed no significant associations with cognitive function (CERAD WLLT *p* = .335, WLLRT *p* = .786, WLLT-IC *p* = .612 and WLLRT-IC *p* = .774, AFT *p* = .321, and DSST *p* = .187; Supplementary Table S1).

**Table 2.** Multiple Linear Regression Analysis of the Association Between Bioavailable Testosterone and Cognitive Function by Test Cognitive Performance

Cognitive Function	β	*p*	*R* ^2^
CERAD WLLT	0.008	.132	.130
CERAD WLLRT	0.001	.643	.128
CERAD WLLT-IC	0.000	.979	.068
CERAD WLLRT-IC	0.001	.387	.055
AFT	0.013	.057	.270
DSST	0.049	.002	.338

*Notes:* AFT = Animal Fluency Test; CERAD = Consortium to Establish a Registry for Alzheimer’s Disease; CI = confidence interval; DSST = Digit Symbol Substitution Test; WLLT = Word List Learning Test; WLRT = Word List Recall Test. WLLT-IC = Word List Learning Test—Intrusion Word Count; WLRT-IC = Word List Recall Test—Intrusion Word Count.

## Discussion

The current study explored the association between calculated bioavailable testosterone and cognitive function in older men living in the United States. Our results demonstrated a significant association between calculated bioavailable testosterone with DSST score, after adjustment for multiple sociodemographic, anthropometric, and nutritional covariates. The effect was insignificant on CERAD WLLT, WLLT-IC, WLRT and WLRT-IC, and AFT scores.

Our study adds to the existing body of literature that supports the potential role of testosterone for regulating cognitive function in men. For example, it has been previously shown that calculated bioavailable testosterone in healthy men 20–84 years old is significantly associated with decrements in visual and auditory learning (21). Likewise, a significantly, albeit weak negative correlation (*r* = −.222) was found in older men 55 years old and above, between measured bioavailable testosterone and executive function via trails B test (22). Furthermore, Chu et al. presented that measured bioavailable testosterone levels in older Chinese men with amnestic mild cognitive impairment and Alzheimer’s disease aged 55–93 years were positively associated with delayed Stroop word recall tests and verbal and visual memory (23). Additionally, further correlations were observed in independently living men aged 40–80 years with calculated bioavailable testosterone and improved processing speed and executive function (24). Similarly, in healthy older men above 65 years of age, calculated bioavailable testosterone was associated with trails B and digit symbol tests that measured several indices of cognitive function, including attention, working memory, psychomotor performance, and perpetual organization (25).

In contrast to our findings, several studies fail to report positive associations between testosterone and cognitive function. Geerlings et al. revealed that in older Japanese American men aged 71–93 years without dementia, calculated bioavailable testosterone using quantitative competitive immunoassay—an inferior assessment method compared to LC–MS—across serum concentration tertiles did not have any association with Cognitive Abilities Screening Instrument score (26). The latter is a quantitative assessment of attention, concentration, orientation, short- and long-term memory, language and visual construction, verbal fluency, abstraction, and judgment (27). Likewise, Alibhai et al. reported that high concentrations of calculated bioavailable testosterone in older men with or without prostate cancer aged 50–87 years old were not linked with processing speed (trails A and Delis–Kaplan executive function system color-word interference test), attention (digit and spatial span forward score), verbal fluency (controlled oral word association and animal fluency test), visuospatial abilities (card rotation and judgment of line orientation raw scores), and working memory (conditional associative learning test) (28). Evidence of a nonsignificant association between measured bioavailable testosterone levels and measures of cognition such as total memory interference in community-dwelling men aged 35–80 years has also been reported (29).

Our findings, combined with those of previous studies, may appear conflicting; however, the varied methodological approaches adopted offer potential explanations to these discrepancies. For instance, the variability among studies, in part, may be explained by the complexity of different cognitive measures and assessment tools used for the assessment of indices of cognition. The score for the one test we have found significant associations with calculated bioavailable testosterone is characterized by its brevity and high sensitivity for identifying individuals with cognitive impairments. The DSST has particularly shown to be sensitive at detecting both cognitive dysfunction as well as changes in cognitive dysfunction in a range of clinical populations (30). In addition, multiple studies recruited participants with a wide aging range that may be a confounder in assessing the relationship of calculated or measured bioavailable testosterone in aging cohorts similar to our study (60 years and above). The conflicting literature review findings can also be attributed to the method of testosterone assay (eg, immunoassay vs LC/MS) used. For example, the precision and accuracy of immunoassay methods have recently been questioned, particularly when assessing low levels of testosterone (31), a characteristic associated with aging. Indeed, at the lower range of testosterone, up to 40% of results from immunoassay vary by more than 20% from the reference assay result derived via gold-standard LC/MS–MS approaches (31). As such, testosterone values derived by LC/MS are the preferred standard in combination with SHBG and albumin to derive bioavailable or active testosterone. Moreover, in our analyses, sleep duration was one of the most important covariates that can influence concentration of bioavailable testosterone. It has been shown that the amount of nighttime sleep measured by polysomnography is an independent predictor of participants’ morning total and free testosterone levels (32). Additionally, 7 days of sleep restriction (5 hours of sleep per night) in younger men results in a decrease of daytime testosterone levels by ~10%–15% (33). In most studies that have explored the associations between calculated/measured bioavailable testosterone (21–26,28,29), sleep duration was not accounted for as a covariate, and therefore, this may be a potential explanation for discrepancies in existing literature. In our case, the inclusion of sleep duration as a covariate may have contributed to a large decrease in our sample size; however, the confidence in our data and analyses were consolidated by the inclusion of sleep duration. Finally, another strength of our study is the statistical analyses employed, whereby we explored the linear relationship between testosterone and cognitive performance rather than investigating the prediction risk (odds ratio) of cognitive performance based on testosterone levels as others have done (12). Estimates from linear models explore the whole distribution of a variable rather than dichtomizing the data into above/below thresholds. This is more insightful, as from a linear regression model, one can predict the magnitude of change, as opposed to simply its occurrence.

Therefore, different methods of measuring bioavailable testosterone, different age ranges, covariates, statistical analyses, and assessment tools of cognitive performance among studies may have accounted for the differential associations of bioavailable testosterone with cognitive function in older men.

Undoubtedly, further research is needed to improve our understanding of this potential causal link given that our limited interpretations are based on the scarcity of literature on how testosterone may alter brain physiology and how that translates to changes in cognitive performance. For instance, testosterone may influence spatial memory by increasing the neuron volume of rostral hippocampus (34), changes that may be attributed to the direct effect of androgen receptors in the hippocampus (35) and their influence on synaptic plasticity (36) since it has been shown that long-term potentiation–like cortical plasticity impairment is a key phenomenon in Alzheimer’s patients (37), while this impairment correlates with less-efficient verbal memory (37). Furthermore, another explanation underlies the increased levels of neurotrophines, including the nerve growth factor in the hippocampus and the upregulation of its receptor by forebrain neurons (38), along with the depolarization of *N*-methyl-d-aspartate receptor in hippocampal pyramidal cells and regeneration of its neurotransmitter-evoked actions (39). Prospective studies specifically designed to confirm the influence of bioavailable testosterone at different serum levels on cognitive function and its domains are warranted, particularly longitudinal studies to monitor changes in testosterone throughout life. Such approaches, though logistically challenging, will undoubtedly provide novel insights into the potential role of an age-related decline in testosterone levels on cognitive function. Improving our understanding of the impacts of testosterone on cognitive function in older age may potentially have a meaningful impact on the development of therapeutic interventions to improve patient treatment and outcome and extending the health span.

### Strengths and Limitations

Our retrospective analysis study employed large nationally representative data (ie, NHANES) that have been subjected to rigorous quality control. In addition, multiple covariates were adjusted for during analysis to accurately examine (and isolate) the association between bioavailable testosterone and cognitive function in older adults. In doing so, overnight fasted concentrations of bioavailable testosterone, as opposed to total testosterone, were utilized in exploring the relationship between testosterone and cognitive function, overcoming the interacting age-related effect of testosterone by SHBG and albumin levels (40).

However, it is noteworthy that our study also had several limitations. Observational studies using a cross-sectional model are unable to reveal a cause-and-effect relationship between dependent and independent variables. Additionally, mood state could have affected mental factors accounting for changes in cognitive performance that may not be directly linked with total and/or bioavailable testosterone levels. Testosterone may alter amygdala activity and connectivity, reducing functional coupling with the orbitofrontal cortex during face judgment tasks, and increasing during in response to emotional face tasks (41–43). Finally, cognitive function tests collected from NHANES focused on aspects of cognitive performance and may not fully represent overall cognitive function given the multiple mental parameters and assessment tools are warranted. For instance, backward number recall test may stimulate the parietal, occipital, frontal, and temporal cortices to a greater extend as opposed to forward recall testing (44).

## Conclusion

In a population of older adults over the age of 60, calculated bioavailable testosterone was significantly associated with specific indices of cognitive function such as processing speed, but showed no effect on learning, verbal fluency and episodic memory, and memory intrusion. The equivocal nature of our understanding around the impacts of testosterone on cognitive functions lends to the notion for the need for more prospective studies specifically designed to confirm the influence of bioavailable testosterone at different serum levels on cognitive function and its domains.

## Supplementary Material

glac162_suppl_Supplementary_Table_S1

## Data Availability

The data sets analyzed in this study can be accessed in the 2013–2014 National Health and Nutrition Examination Survey (NHANES; https://www.cdc.gov/nchs/nhanes/index.htm).
