# Supplementary material for: Associations of Bioavailable Serum Testosterone With Cognitive Function in Older Men: Results From the National Health and Nutrition Examination Survey
Source: J Gerontol A Biol Sci Med Sci. 2022 Aug 5;78(1):151–7. doi: 10.1093/gerona/glac162 (PMC9879757; doi:10.1093/gerona/glac162)
Supplement: glac162_suppl_Supplementary_Table_S1 [file glac162_suppl_supplementary_table_s1.docx]

**Supplementary Material**

| **Table S1.** Multiple linear regression analysis of the association between total testosterone and cognitive function by test cognitive performance. | | | |
| --- | --- | --- | --- |
| **Cognitive Function** | **β** | ***P*** | ***R*^2^** |
| CERAD WLLT | 0.002 | 0.335 | 0.124 |
| CERAD WLLRT | 0.000239 | 0.786 | 0.128 |
| CERAD WLLT-IC | -0.000234 | 0.612 | 0.069 |
| CERAD WLLRT-IC | 0.000091 | 0.774 | 0.052 |
| AFT | 0.002 | 0.321 | 0.260 |
| DSST | 0.007 | 0.187 | 0.310 |
| **Abbreviations:**  AFT: Animal Fluency Test, CERAD: Consortium to Establish a Registry for Alzheimer's Disease, CI: Confidence Interval, DSST: Digit Symbol Substitution Test, WLLT: Word List Learning Test, WLRT: Word List Recall Test. WLLT-IC: Word List Learning Test – Intrusion , WLRT-IC: Word List Recall Test – Intrusion . | | | |
